# Increased isobutanol production in *Saccharomyces cerevisiae *by overexpression of genes in valine metabolism

**DOI:** 10.1186/1754-6834-4-21

**Published:** 2011-07-28

**Authors:** Xiao Chen, Kristian F Nielsen, Irina Borodina, Morten C Kielland-Brandt, Kaisa Karhumaa

**Affiliations:** 1 Center for Microbial Biotechnology, Department of Systems Biology, Technical University of Denmark, Søltofts Plads, Building 223, DK-2800 Kgs, Lyngby, Denmark

## Abstract

**Background:**

Isobutanol can be a better biofuel than ethanol due to its higher energy density and lower hygroscopicity. Furthermore, the branched-chain structure of isobutanol gives a higher octane number than the isomeric *n*-butanol. *Saccharomyces cerevisiae *was chosen as the production host because of its relative tolerance to alcohols, robustness in industrial fermentations, and the possibility for future combination of isobutanol production with fermentation of lignocellulosic materials.

**Results:**

The yield of isobutanol was improved from 0.16 to 0.97 mg per g glucose by simultaneous overexpression of biosynthetic genes *ILV2, ILV3*, and *ILV5 *in valine metabolism in anaerobic fermentation of glucose in mineral medium in *S. cerevisiae*. Isobutanol yield was further improved by twofold by the additional overexpression of *BAT2*, encoding the cytoplasmic branched-chain amino-acid aminotransferase. Overexpression of *ILV6*, encoding the regulatory subunit of Ilv2, in the *ILV2 ILV3 ILV5 *overexpression strain decreased isobutanol production yield by threefold. In aerobic cultivations in shake flasks in mineral medium, the isobutanol yield of the *ILV2 ILV3 ILV5 *overexpression strain and the reference strain were 3.86 and 0.28 mg per g glucose, respectively. They increased to 4.12 and 2.4 mg per g glucose in yeast extract/peptone/dextrose (YPD) complex medium under aerobic conditions, respectively.

**Conclusions:**

Overexpression of genes *ILV2, ILV3, ILV5*, and *BAT2 *in valine metabolism led to an increase in isobutanol production in *S. cerevisiae*. Additional overexpression of *ILV6 *in the *ILV2 ILV3 ILV5 *overexpression strain had a negative effect, presumably by increasing the sensitivity of Ilv2 to valine inhibition, thus weakening the positive impact of overexpression of *ILV2, ILV3*, and *ILV5 *on isobutanol production. Aerobic cultivations of the *ILV2 ILV3 ILV5 *overexpression strain and the reference strain showed that supplying amino acids in cultivation media gave a substantial improvement in isobutanol production for the reference strain, but not for the *ILV2 ILV3 ILV5 *overexpression strain. This result implies that other constraints besides the enzyme activities for the supply of 2-ketoisovalerate may become bottlenecks for isobutanol production after *ILV2, ILV3*, and *ILV5 *have been overexpressed, which most probably includes the valine inhibition to Ilv2.

## Background

Environmentally friendly production of biofuels is a target of great interest due to climate change and the need for renewable transportation fuels. Microbial production of chemicals to be used as liquid biofuels will allow the use of renewable raw materials such as lignocellulose. Optimally, biomass from agricultural and forestry waste products could be used as raw material.

Bioethanol is the most well established biofuel, with existing commercial production. Several pilot plants are in operation for lignocellulosic ethanol production in several countries. However, the chemical properties of ethanol, such as a high tendency to absorb water, are not optimal for all purposes. For many purposes, such as a jet fuel, improved properties are required with regard to hygroscopicity and energy density. Higher alcohols, such as *n*-butanol and isobutanol, represent possible alternatives. Compared with *n*-butanol, isobutanol has the advantage of having a higher octane number, and the possibility of usage outside the fuel industry as well [1].

Microbial production of isobutanol has been studied in food fermentations and alcoholic beverages for flavour profiling since the 1970s [2,3]. In recent years, production of isobutanol has been investigated as a biofuel in engineered *Escherichia coli *to reach a concentration of 22 g/l during aerobic cultivations [4]. However, as isobutanol concentrations over 15 g/l are toxic to *E. coli *[5], this concentration may also be close to the maximal possible production in this bacterium. Since *Corynebacterium glutamicum *is more tolerant to isobutanol than is *E. coli*, it was engineered as a host and produced about 4 g/l of isobutanol in the presence of oxygen [5]. Yeast is well known to be tolerant to alcohols. *Saccharomyces cerevisiae *is tolerant to up to 20% ethanol [6]. In a test of many microbial strains for butanol tolerance, baker's yeast *S. cerevisiae *and *Lactobacilli *were the only microbes able to grow in butanol concentrations higher than 20 g/l [7]. In addition, yeast is relatively robust in other respects, commonly used in fermentations with harsh conditions, and its outstanding performance in lignocellulosic hydrolysates would enable future combination of the higher alcohol production with fermentation of lignocellulosic materials. Yeast naturally produces small amounts of higher alcohols through the degradation of amino acids [8,9], such as isobutanol among others [2,3]. These are of great importance in the flavour profiles of beer and wine, as they generate desired fruity aromas in these products when produced in favourable amounts. Use of a host that naturally produces isobutanol may in principle offer an opportunity to avoid the use of heterologous pathways, which often require extensive work for optimal expression. Because of all these advantages, *S. cerevisiae *is a potential alternative host for higher alcohol production.

**Figure 1:**
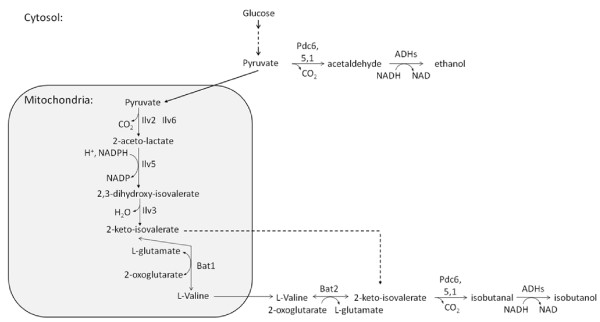
**The metabolic pathways from pyruvate to isobutanol and ethanol in *Saccharomyces cerevisiae***. The enzymes that catalyse the pathway from pyruvate to L-valine in the mitochondria are: acetolactate synthase (Ilv2+Ilv6), acetohydroxyacid reductoisomerase (Ilv5), dihydroxyacid dehydratase (Ilv3), and branched-chain amino-acid aminotransferase (Bat1). Ilv2 is the catalytic subunit of acetolactate synthase, and Ilv6 is the regulatory subunit. The enzymes that catalyse the pathway from L-valine to isobutanol in the cytosol are pyruvate decarboxylases (Pdc6, 5, 1) and alcohol dehydrogenases (ADHs). Pyruvate decarboxylases and alcohol dehydrogenases also catalyse the pathway from pyruvate to ethanol in *S. cerevisiae*. The broken line indicates the export of 2-ketoisovalerate from mitochondria to cytosol.

We studied the effect of concomitant overexpression of several structural genes for the endogenous yeast valine biosynthetic pathway (Figure 1) for anaerobic isobutanol production in *S. cerevisiae*. It is well studied how yeast can convert valine to isobutanol [9], thereby isobutanol is a side product of valine synthesis, and our interest was whether increased flux in the valine biosynthetic pathway would increase isobutanol production. Isobutanol can be produced from pyruvate via formation of 2-ketoisovalerate (Figure 1). First, acetolactate synthase (Ilv2 + Ilv6) converts two molecules of pyruvate to 2-acetolactate, which is then reduced to 2,3-dihydroxyisovalerate by acetohydroxyacid reductoisomerase (Ilv5). Further, 2,3-dihydroxyisovalerate is converted to 2-ketoisovalerate by dihydroxyacid dehydratase (Ilv3). Ilv6 is the regulatory subunit of acetolactate synthase and an enhancer of Ilv2 catalytic activity. The bidirectional reaction between 2-ketoisovalerate and valine is catalysed by the branched-chain amino-acid aminotransferases (Bat1 and Bat2, present in the mitochondrial matrix and the cytosol, respectively). Finally, the 2-ketoisovalerate is converted to isobutanol by pyruvate decarboxylases (PDC) and alcohol dehydrogenases (ADH). We have here used overexpression of the valine pathway genes *ILV2, ILV3 *and *ILV5 *to generate a sixfold higher isobutanol yield. The finding that the isobutanol production in yeast that ferments a rich, complex medium could be increased by overexpression of Bat2 [10] prompted us to overexpress *BAT2 *additional to the overexpression of *ILV2, ILV3 *and *ILV5 *in synthetic medium. This turned out to increase isobutanol production further by twofold. However, overexpression of *ILV6*, encoding the regulatory subunit of the first committed step in valine biosynthesis, weakened the impact of overexpression of *ILV2, ILV3*, and *ILV5 *on isobutanol production. After the initiation of the present work, the results of somewhat similar genetic approaches on isobutanol production in *S. cerevisiae *appeared in patent applications [11,12], but not in the scientific literature.

## Results and discussion

The objective of this work was to study isobutanol formation in *S. cerevisiae*, and to improve isobutanol production by metabolic engineering. We studied overexpression of the genes *ILV2, ILV3, ILV5, ILV6*, and *BAT2*, involved in valine metabolism, in different combinations, and investigated the isobutanol production in the constructed strains.

### Improvement of anaerobic isobutanol production by overexpression of genes in valine metabolism

To overexpress the genes, *ILV2, ILV3*, and *ILV5*, which encode the catalysts for the conversion of pyruvate to 2-ketoisovalerate (direct precursor of L-valine), the coding regions were fused with the *S. cerevisiae PGK1 *promoter. The resulting PGK1-ILV2, PGK1-ILV3 and PGK1-ILV5 DNA fusion fragments were cloned into integration vectors YDp-L, YDp-W and YDp-H [13], respectively. The three resulting plasmids were linearised by cleavage of the *ILV *genes and successively integrated into the genome of CEN.PK 2-1C (*MAT*α *leu2-3, 112 his3-Δ1 ura3-52 trp1-289 MAL2-8*(Con) *MAL3 SUC3*) (a kind gift of P Kötter, Goethe Universität Frankfurt, Frankfurt, Germany The resulting strain was designated ILV235_XCY561 (*ILV2 ILV3 ILV5 *overexpression strain). Successful genomic integration was confirmed by analytical polymerase chain reaction (PCR) on chromosomal DNA. The copy number of the integrated gene was estimated by performing quantitative real-time PCR with genomic DNA as template [14,15]. The estimates of the copy numbers of *ILV2, ILV3 *and *ILV5 *in ILV235_XCY561 given by the quantitative PCR were 4.1 ± 0.90, 2.1 ± 0.65 and 3.6 ± 1.12 times larger than those in the reference strain, respectively. Multiple integrations may have happened in some cases. The expression of *ILV2, ILV3 *and *ILV5 *at the transcriptional level in ILV235_XCY561 was also measured by quantitative real-time PCR. The mRNA levels of *ILV2, ILV3 *and *ILV5 *in ILV235_XCY561 measured by quantitative (qPCR) were 6.42 ± 2.20, 9.98 ± 1.31 and 3.24 ± 0.61 times larger than those in the reference strain, respectively. Overexpression of these three genes at mRNA level was proved. However, no enzymatic assays were performed to confirm the overexpression since the reagents needed are not commercially available.

**Figure 2:**
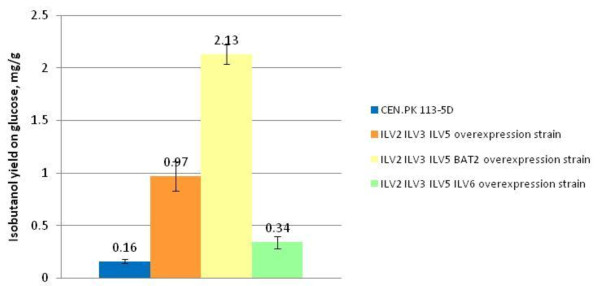
**Effects of overexpression of *ILV *genes on isobutanol yield under anaerobic conditions**. Isobutanol yields (mg per g glucose) of the reference strain (CEN.PK 113-5D) and the *ILV2 ILV3 ILV5*, the *ILV2 ILV3 ILV5 BAT2*, and the *ILV2 ILV3 ILV5 ILV6 *overexpression strains were presented with columns with different colours, and the values are shown on the tops of each column. All cultivations were carried out in fermenters in mineral medium with 40 g glucose/l under anaerobic conditions.

The *ILV2 ILV3 ILV5 *overexpressing strain (ILV235_XCY561) and the reference strain (CEN.PK 113-5D) were cultivated in mineral glucose medium supplemented with uracil in fermenters under anaerobic conditions. Primary metabolite concentrations were measured by high-performance liquid chromatography (HPLC) throughout the fermentation. Isobutanol concentration was measured by gas chromatography (GC) only from the samples taken at the end of fermentations after glucose depletion, due to the large sample volume needed for reliable measurements. The *ILV2 ILV3 ILV5 *overexpression strain produced 0.97 ± 0.14 mg isobutanol per g glucose, which was sixfold higher than the reference strain (Figure 2). No difference was detected between the *ILV2 ILV3 ILV5 *overexpression strain and the reference strain in the production of biomass, ethanol, pyruvate, succinate, glycerol, acetate and CO_2 _(Table 1). Our interpretation is that overexpression of gene *ILV2, ILV3*, and *ILV5 *led to a higher concentration of 2-ketoisovalerate, which resulted in higher isobutanol production.

**Table 1:** Effects of gene overexpression on growth rates and product yields under anaerobic batch fermentations^a^

Strain	Reference strain^b^	*ILV2 ILV3 ILV5 *overexpression strain^b^	*ILV2 ILV3 ILV5 BAT2 *overexpression strain^b^	*ILV2 ILV3 ILV5 ILV6 *overexpression strain^b^
Specific growth rate, per h	0.38 ± 0.02	0.29 ± 0.02	0.16 ± 0.01	0.18 ± 0.00

Biomass (CH_1.78_O_0.6_N_0.19_) yield, g/g glucose	0.10 ± 0.00	0.11 ± 0.01	0.07 ± 0.01	0.11 ± 0.00

Ethanol yield, g/g glucose	0.38 ± 0.01	0.37 ± 0.02	0.34 ± 0.01	0.37 ± 0.00

Pyruvate yield, g/g glucose	0.0011 ± 0.007	0.0016 ± 0.0007	0.0032 ± 0.0006	0.0028 ± 0.0001

Succinate yield, g/g glucose	0.0016 ± 0.0006	0.0006 ± 0.0001	0.0028 ± 0.0001	0.0035 ± 0.0003

Glycerol yield, g/g glucose	0.100 ± 0.008	0.087 ± 0.006	0.095 ± 0.001	0.099 ± 0.003

Acetate yield, g/g glucose	0.0076 ± 0.0014	0.0088 ± 0.0010	0.0110 ± 0.0006	0.0069 ± 0.0003

CO_2 _yield, g/g glucose	0.32 ± 0.01	0.39 ± 0.01	0.37 ± 0.04	0.41 ± 0.03

Carbon balance deviation,%^b^	5.9 ± 2.2	3.0 ± 4.0	10.9 ± 1.7	1.3 ± 1.7

^a^The mineral medium with 40 g/l of glucose was used.

^b^The reference strain used was CEN.PK 113-5D; The *ILV2 ILV3 ILV5 *overexpression strain used was ILV235_XCY561; The *ILV2 ILV3 ILV5 BAT2 *overexpression strain used were ILV235BAT2_XCY715 and ILV235BAT2_XCY723; The *ILV2 ILV3 ILV5 ILV6 *overexpression strain used was ILV2356_XCY605.

The *ILV2 ILV3 ILV5 *overexpression strain had a maximum specific growth rate of 0.29 ± 0.02 per h, which was slower than the maximum specific growth rate of the reference strain, which was 0.38 ± 0.02 per h. The slower growth rate may be due to a metabolic imbalance caused by the overexpression of the *ILV *genes, which affects some amino acids pools, or due to insufficient expression of the selection markers, which were put at different loci and might not work with the same efficiency as at their wild type locations.

*BAT2 *encodes a branched-chain amino-acid aminotransferase, which catalyses the first reaction in the catabolism of valine in the cytosol [16]. *BAT2 *is highly expressed during stationary phase and repressed during logarithmic growth, which is the opposite way of the regulation of the mitochondrial branched-chain amino-acid aminotransferase, encoded by *BAT1 *[17]. It has been previously shown that overexpression of *BAT2 *alone increases isobutanol concentration in wine fermentations [18]. We therefore decided to investigate whether *BAT2 *overexpression could complement the valine pathway overexpression and result in higher yields and concentrations of isobutanol.

**Figure 3:**
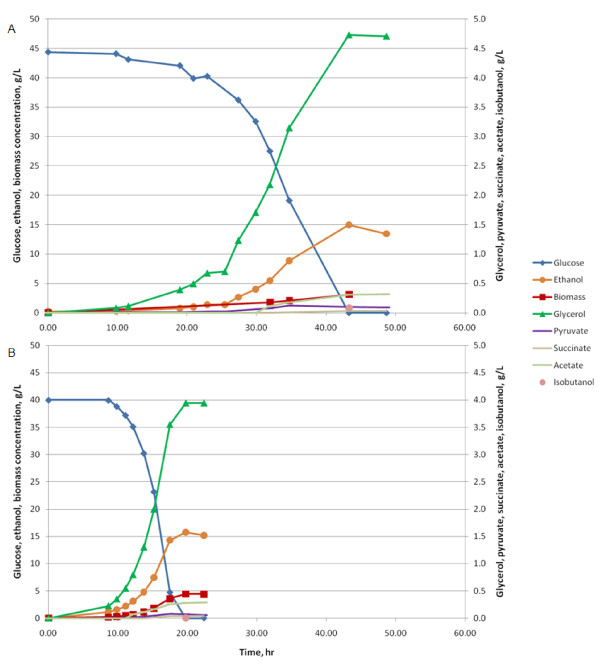
**Time profiles of fermentations of *ILV2 ILV3 ILV5 BAT2 *overexpression (a) and reference strain (b)**. The *ILV2 ILV3 ILV5 BAT2 *overexpression strain and the reference strain CEN.PK 113-5D were cultivated under anaerobic batch fermentations in mineral medium with 40 g glucose/l. The concentrations of glucose, biomass, and products are plotted as a function of time. Isobutanol concentrations were measured after glucose depletion in both cases. Fermentations were performed in triplicate, representative cultivations are shown.

An *ILV2 ILV3 ILV5 BAT2 *overexpression strain, ILV235BAT2_XCY715, was thus constructed by using the same promoter and the same molecular strategy. The estimate of the copy number of gene *BAT2 *in ILV235BAT2_XCY715 was 1.5 ± 0.50 times larger than the one in the reference strain, which was in accordance with the expected copy number of two. The overexpression of *BAT2 *at the transcriptional level in ILV235BAT2_XCY715 was 90.82 ± 0.45 times larger than that in the reference strain. The different overexpression level among *ILV2, ILV3, ILV5 *and *BAT2*, reflects the fact that expression level controlled by inserted promoters depends on the surrounding sequence. Anaerobic fermentations with ILV235BAT2_XCY715 were carried out in fermenters under the conditions described above. The GC results showed that the *ILV2 ILV3 ILV5 BAT2 *overexpression strain produced 13 times more isobutanol per g glucose than the reference strain. This is an improvement of twofold over that of the *ILV2 ILV3 ILV5 *overexpression strain (Figure 2). The biomass yield on glucose of the *ILV2 ILV3 ILV5 BAT2 *overexpression strain was 0.07 ± 0.01 g per g glucose, which was lower than the biomass yields of the *ILV2 ILV3 ILV5 *overexpression strain and the reference strain, which were 0.10 ± 0.00 and 0.11 ± 0.01, respectively. A carbon balance calculation showed that after accounting for carbon in produced biomass, isobutanol, ethanol, pyruvate, succinate, glycerol, acetate and CO_2_, there was about 11% of carbon missing (Table 1). We have investigated whether the missing carbon could be explained by the increase in the production of 3-methylbutanol and 2-methylbutanol, since the *BAT2 *encoding aminotransferase also catalyses the first step in the leucine and isoleucine catabolism pathways, which produce 3-methylbutanol and 2-methylbutanol, respectively [9,19,20]. GC analysis showed that the yield of 3-methylbutanol increased from 0.28 to 0.48 mg per g glucose (0.0330% carbon) compared to the reference strain, and there was no increase observed in 2-methylbutanol yield. These increases, however, were not large enough to explain the missing carbon. The deviation in carbon balance could be due to some other products not included in the present analysis. The time profiles of anaerobic fermentations of the *ILV2 ILV3 ILV5 BAT2 *overexpression strain and the CEN.PK 113-5D reference strain are presented in Figure 3a, b, respectively. Glucose consumption and all measured production rates were much higher in the reference strain than in the *ILV2 ILV3 ILV5 BAT2 *overexpression strain. The maximum specific growth rate of the *ILV2 ILV3 ILV5 BAT2 *overexpression strain was 0.16 ± 0.01 per h, and thus much lower than that of the reference strain and the *ILV2 ILV3 ILV5 *overexpression strain, which has the growth rate at 0.38 ± 0.02 and 0.29 ± 0.02 per h, respectively. The decrease in growth rate could be due to the same reasons as explained earlier for the slow growth rate of the *ILV2 ILV3 ILV5 *overexpression strain. The very high overexpression of *BAT2 *might result in a further metabolic imbalance of some amino acids pools, and could be the cause the further drop of the growth rate of the *ILV2 ILV3 ILV5 BAT2 *overexpression strain. An independent transformant of the *ILV2 ILV3 ILV5 *overexpression strain with the *BAT2 *overexpression construct, named ILV235BAT2_XCY723, was investigated, and it behaved identically to ILV235BAT2_XCY715.

Ilv6 is the regulatory subunit of acetolactate synthase and has been described as an enhancer of Ilv2 catalytic activity. To investigate whether *ILV6 *overexpression would further improve isobutanol production by possibly approaching equimolar amounts of both proteins, *ILV6 *was overexpressed in the strain background with *ILV2, ILV3 *and *ILV5 *overexpression. The resulting strain was designated ILV2356_XCY605, here also referred to as the *ILV2 ILV3 ILV5 ILV6 *overexpression strain. The estimate of the copy number of *ILV6 *in ILV2356_XCY605 was 2.8 ± 1.67 times larger than that in the reference strain. We also estimated the copy number of *ILV6 *in ILV235BAT2_XCY715 and that of *BAT2 *in ILV2356_XCY605. Their copy numbers were 0.9 ± 0.31 and 1.0 ± 0.42 times, respectively, compared to the reference strain, which was in agreement with the fact that these two genes were not overexpressed in their respective strains. The overexpression of *ILV6 *at the transcriptional level in ILV2356_XCY605 was 8.07 ± 1.14 times larger than that in the reference strain. Unexpectedly, integration of the overexpression construct with *ILV6 *caused threefold less isobutanol production, namely 0.34 mg per g glucose (Figure 2), and a drop of the maximum specific growth rate to 0.18 ± 0.00 per h. The yields of the other measured products, that is, biomass, ethanol, pyruvate, succinate, glycerol, acetate and CO_2 _were largely unaffected (Table 1). We interpret the lower yield of isobutanol upon overexpression of *ILV6 *to be due to an increased sensitivity of Ilv2 to valine inhibition, weakening the positive impact of overexpression of *ILV2, ILV3*, and *ILV5 *on increasing isobutanol production in *S. cerevisiae*, since Ilv6 stimulates Ilv2 catalytic activity sevenfold to tenfold and confers Ilv2 sensitivity to valine inhibition [21,22].

### Influence of various media on isobutanol production and growth rate

The *ILV2 ILV3 ILV5 *overexpression strain and the CEN.PK 113-5D reference strain were cultivated under aerobic conditions in shake flasks to further investigate the effect of valine pathway overexpression on growth rates and isobutanol production. Buffered mineral medium with 40 g glucose/l and yeast extract/peptone/dextrose (YPD) complex medium with 17 g glucose/l were used. The growth rates and the main product yields are shown in Table 2. In the mineral medium, the *ILV2 ILV3 ILV5 *overexpression strain had a threefold lower maximum specific growth rate (0.110 per h) than the reference strain (0.359 per h). In YPD, however, they grew at similar growth rates, about 0.5 per h. Our interpretation is that genetic manipulations in the *ILV2 ILV3 ILV5 *overexpression strain might cause a metabolic imbalance, which perhaps affected some amino acid pools, or the improper functioning of the selection markers. Any of these problems could be compensated for by growing the overexpression strain in the complex medium.

**Table 2:** Aerobic batch cultivations of the *ILV2 ILV3 ILV 5 *overexpression strain and reference strain in shake flasks^a^

	Buffered mineral medium		YPD complex medium	
	
	Reference stain^b^	*ILV2 ILV3 ILV5 *overexpression strain^b^	Reference strain	*ILV2 ILV3 ILV5 *overexpression strain
Specific growth rate, per h	0.36 ± 0.00	0.11 ± 0.00	0.52 ± 0.00	0.50 ± 0.00

Biomass (CH_1.78_O_0.6_N_0.19_) yield, g/g glucose	0.33 ± 0.06	0.14 ± 0.05	0.34 ± 0.00	0.35 ± 0.00

Ethanol yield, g/g glucose	0.22 ± 0.05	0.28 ± 0.10	0.12 ± 0.01	0.19 ± 0.02

Pyruvate yield, g/g glucose	0.0002 ± 0.0000	0.0002 ± 0.0000	0.0137 ± 0.0003	0.0134 ± 0.0005

Succinate yield, g/g glucose	0.20 ± 0.02	0.26 ± 0.01	0.02 ± 0.00	0.01 ± 0.00

Glycerol yield, g/g glucose	0.006 ± 0.006	0.026 ± 0.017	0.002 ± 0.000	0.013 ± 0.000

Acetate yield, g/g glucose	0.0000 ± 0.0000	0.0052 ± 0.0022	0.0929 ± 0.0047	0.0929 ± 0.0018

^a^The cultivations were carried out in shake flask. Buffered mineral medium containing 40 g/l glucose and YPD complex medium containing 17 g/l glucose were used.

^b^The reference strain used was CEN.PK 113-5D. The overexpression strain used was ILV235_XCY561.

YPD = yeast extract/peptone/dextrose.

**Figure 4:**
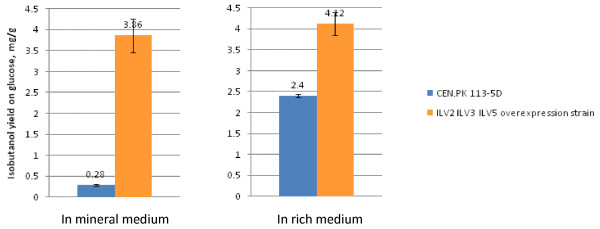
**Effects of gene overexpression on isobutanol yield in various media under aerobic conditions**. The isobutanol yields (mg per g glucose) of the reference strain (CEN.PK 113-5D) and the *ILV2 ILV3 ILV5 *overexpression strains are presented with columns with different colours, and the values are shown on the top of each column. All cultivations were carried out aerobically in shake flasks in either mineral medium with 40 g glucose/l or yeast extract/peptone/dextrose (YPD) complex medium with 17 g glucose/l.

Isobutanol yields of the *ILV2 ILV3 ILV5 *overexpression strain and the reference strain under aerobic cultivation conditions in different media are compared in Figure 4. In mineral medium the *ILV2 ILV3 ILV5 *overexpression strain produced 3.86 mg isobutanol per g glucose, and the reference strain produced 0.28 mg isobutanol per g glucose. In YPD complex medium the *ILV2 ILV3 ILV5 *overexpression strain and the reference strain produced 4.12 and 2.4 mg isobutanol per g glucose, respectively. There were 2.12 mg isobutanol per g glucose of increase for the reference strain, and only 0.26 mg isobutanol per g glucose of increase for the *ILV2 ILV3 ILV5 *overexpression strain. We interpret this due to both overexpression of *ILV2, ILV3*, and *ILV5 *and uptake of valine from the complex medium caused a strong increase of the pool of valine, and thereby an increase of isobutanol production. Since the provision of valine in the medium by using the YPD complex medium did not increase the isobutanol production yield of the *ILV2 ILV3 ILV5 *overexpression strain much, it appears that other constraints besides the enzyme activities for the supply of 2-ketoisovalerate in the *ILV2 ILV3 ILV5 *overexpression strain might have become bottlenecks. These could include valine inhibition to Ilv2, transportation of 2-ketoisovalerate or valine from mitochondria to cytosol, and affinities of PDCs to 2-ketoisovalerate in cytosol.

In this study, the isobutanol production yield was increased first by simultaneously overexpression genes, *ILV2, ILV3 *and *ILV5 *in valine biosynthetic pathway. However, which gene(s) out of these three is (are) needed to reach the same increased level of isobutanol yield, was not investigated in this study. Even though the isobutanol production yield was improved by ninefold by overexpression of *ILV2, ILV3, ILV5*, and *BAT2*, in valine biosynthetic and degradation pathways, the isobutanol production yield was still too low for commercial applications. The low production of isobutanol was probably due to the regulation control of valine production in mitochondria, which limited the flux toisobutanol. Moving the pathway from pyruvate to 2-ketoisovalerate from mitochondria to cytosol would benefit the production of isobutanol in yeast.

## Conclusions

Isobutanol production yield on glucose was first increased from 0.16 to 0.97 mg isobutanol per gram of glucose by overexpression of genes *ILV2, ILV3 *and *ILV5*, which encode the catalysts for the conversion of pyruvate to 2-ketoisovalerate, the immediate precursor of valine. With the background of *ILV2 ILV3 ILV5 *overexpression, isobutanol yield was further improved twofold by overexpression of *BAT2*, encoding the catalyst for the first step of valine catabolism. Also with the background of the *ILV2 ILV3 ILV5 *overexpression, overexpression of *ILV6*, encoding a regulatory subunit that combines with Ilv2, which catalyses the first committed step in valine biosynthesis, resulted in a threefold drop of the yield of isobutanol. We interpret this to be due to an increased sensitivity of Ilv2 to valine inhibition, weakening the positive impact of overexpression of *ILV2, ILV3*, and *ILV5 *on increasing isobutanol production.

Aerobic cultivation of the *ILV2 ILV3 ILV5 *overexpression strain and the reference strain in shake flasks indicated that uptake of valine has the same effect on increasing the valine pool and thereby isobutanol production as on increasing the flux capacity from pyruvate to 2-ketoisovalerate by overexpression of the three genes. Thus, the enzyme activities for the supply of 2-ketoisovalerate were not a bottleneck any longer in the *ILV2 ILV3 ILV5 *overexpression strain. Other bottlenecks, such as the regulation of valine production, export of valine or 2-ketoisovalerate from mitochondria to cytosol, or the affinities of PDCs to 2-ketoisovalerate in cytosol, should be investigated in order to further increase the yield of isobutanol.

## Methods

### Media and culture conditions

The mineral medium, which was used in all anaerobic fermentations, had the following composition (per litre): (NH_4_)_2_SO_4_, 10 g; KH_2_PO_4_, 6 g; MgSO_4_·7H_2_O, 1 g; Antifoam 289 (A-5551; Sigma-Aldrich, St Louis, MO, USA), 0.2 ml; trace metal solution, 2 ml; vitamin solution, 2 ml; and ergosterol solution, 2 ml. This medium was supplemented with 40 g/l glucose and 0.1 g/l uracil. The trace metal solution consisted of the following (per litre): ethylenediaminetetraacetic acid (EDTA) (sodium salt), 15.0 g; ZnSO_4_·7H_2_O, 4.5 g; MnCl_2_·2H_2_O, 0.84 g; CoCl_2_·6H_2_O, 0.3 g; CuSO_4_·5H_2_O, 0.3 g; Na_2_MoO_4_·2H_2_O, 0.4 g; CaCl_2_·2H_2_O, 4.5 g; FeSO_4_·7H_2_O, 3 g; H_3_BO_3_, 1 g; and KI, 0.1 g. The vitamin solution contained the following (per litre): biotin, 0.05 g; *p*-aminobenzoic acid, 0.2 g; nicotinic acid, 1 g; calcium pantothenate, 1 g; pyridoxine-HCl, 1 g; thiamine-HCl, 1 g; and *myo*-inositol, 25 g. The ergosterol solution contained 2 g ergosterol and 84 g Tween 80 in 100 ml pure ethanol.

For the precultivations, 50 ml of mineral medium was used in 500 ml shake flasks, with the exception that 0.1 g/l uracil was added when necessary, and no ergosterol was used. The precultures were grown at 30°C with shaking at 200 rpm and a start pH of 5.0.

All aerobic cultivations were performed in 500 ml baffled shake flasks with 100 ml of working volume of either buffered mineral medium or YPD complex medium. Buffered mineral medium contained (per litre): (NH_4_)_2_SO_4_, 15 g; KH_2_PO_4_, 28.8 g; MgSO_4_·7H_2_O, 1 g; Antifoam 289, 0.2 ml; trace metal solution, 4 ml; vitamin solution, 2 ml and 40 g glucose. The compositions of trace metal and vitamin solutions were as above, and 0.1 g/l uracil was used when necessary. The YPD complex medium contained, per litre, 10 g of yeast extract, 20 g of peptone, and 17 g glucose. The aerobic cultivations were carried out at 30°C with shaking at 200 rpm and a start pH of 5.0.

### Strains and strain construction

All *S. cerevisiae *strains used in this study were derivatives of CEN.PK 2-1C strain (*MAT*α *leu2-3, 112 his3-Δ1 ura3-52 trp1-289 MAL2-8*(Con) *MAL3 SUC3*) (Table 3). Strain CEN.PK 113-5D (*MAT*α *SUC2 MAL2-8*^c ^*ura3*-*52*) and CEN.PK 113-7D (*MAT*α *MAL2-8*^c ^*SUC2*), were used as reference strains in fermentation and real-time PCR experiments, respectively. For routine cultivation, YPD solid or liquid medium was used. Synthetic dropout (SD) solid media appropriately lacking uracil or amino acids were used for selection of yeast plasmid transformants. Yeast extract/peptone/glycerol (YPG) glycerol-based solid medium (containing, per litre, 10 g of yeast extract, 20 g of peptone, and 20 g of glycerol) was used for petite test before long-term storage of strains in 20% of glycerol at -80°C.

**Table 3:** Plasmids and strains

Plasmid or strain	Relevant characteristics	Source or reference
Plasmid		

pCR-Blunt II-TOPO	Cloning vector; Km^r^	Invitrogen

YDp-L	pUC9 derivative, with LEU2 marker	[13]

YDp-W	pUC9 derivative, with TRP1 marker	[13]

YDp-H	pUC9 derivative, with HIS3 marker	[13]

YDp-U	pUC9 derivative, with URA3 marker	[13]

pTOPO_P+ILV2	pCR-Blunt II-TOPO with *PGK1 *promoter and *ILV2 *gene from *Saccharomyces cerevisiae *	This study

pTOPO_P+ILV3	pCR-Blunt II-TOPO with *PGK1 *promoter and *ILV3 *gene from *S. cerevisiae *	This study

pTOPO_P+ILV5	pCR-Blunt II-TOPO with *PGK1 *promoter and *ILV5 *gene from *S. cerevisiae*	This study

pTOPO_P+ILV6	pCR-Blunt II-TOPO with *PGK1 *promoter and *ILV6 *gene from *S. cerevisiae *	This study

pTOPO_P+BAT2	pCR-Blunt II-TOPO with *PGK1 *promoter and *BAT2 *gene from *S. cerevisiae *	This study

YDp-L_P+ILV2	Plasmid YDp-L with *PGK1 *promoter and *ILV2*	This study

YDp-W_P+ILV3	Plasmid YDp-W with *PGK1 *promoter and *ILV3*	This study

YDp-H_P+ILV5	Plasmid YDp-H with *PGK1 *promoter and *ILV5*	This study

YDp-U_P+ILV6	Plasmid YDp-U with *PGK1 *promoter and *ILV6*	This study

YDp-U_P+BAT2	Plasmid YDp-U with *PGK1 *promoter and *BAT2*	This study

Strains		

CEN.PK 2-1C	*MAT*α *leu2-3, 112 his3-Δ1 ura3-52 trp1-289 MAL2-8*(Con) *MAL3 SUC3*	P Kötter

CEN.PK 113-5D	*MAT*α *SUC2 MAL2-8*^c ^*ura3*-*52*	P Kötter

CEN.PK 113-7D	*MAT*α *MAL2-8*^c ^*SUC2*	P Kötter

ILV235_XCY561	CEN.PK 2-1C with YDp-H_P+ILV5, YDp-L_P+ILV2, and YDp-W_P+ILV3 inserted into the genome	This study

ILV235BAT2_XCY715, ILV235BAT2_XCY723	CEN.PK 2-1C with YDp-H_P+ILV5, YDp-L_P+ILV2, YDp-W_P+ILV3, and YDp-U_P+BAT2 inserted into the genome.	This study

ILV2356_XCY605	CEN.PK 2-1C with YDp-H_P+ILV5, YDp-L_P+ILV2, YDp-W_P+ILV3, and YDp-U_P+ILV6 inserted into the genome	This study

Throughout strain construction, standard molecular biology methods were used. Primers used for PCR amplification of DNA fragments (*PGK1 *promoter, genes *ILV2, ILV3, ILV5, ILV6 *and *BAT2*) from *S. cerevisiae *are listed in Table 4. Each amplified target gene (*ILV2, ILV3, ILV5, ILV6*, or *BAT2*) was fused to the downstream end of the *PGK1 *promoter sequence (1,480 bp) in subsequent PCR through designed overlapping ends between the promoter and the target gene. All preparative PCRs were performed with Phusion high-fidelity DNA polymerase (Finnzymes, Thermo Fisher Scientific, Vantaa, Finland).

**Table 4:** Primer sequences

Fragment	Primer	Primer sequence (5' to 3')	Restriction site
*PGK1 *promoter	Forward	AAAAAA**CCCGGG**TCTAACTGATCTATCCAAAACTG	*Xma*l

	Reverse	*TGTTTTATATTTGTTGTAAAA*AGTAGA	

*ILV2*	Forward	*TCTACTTTTTACAACAAATATAAAACA*ATGATCAGACAATCTACGCTAA	

	Reverse	AAAAAA**GGCGCC**CAAGCTTGCAATTTTTGACG	*Nar*I

*ILV3*	Forward	*TCTACTTTTTACAACAAATATAAAACA*ATGGGCTTGTTAACGAAAGT	

	Reverse	AAAAAA**GGCGCC**CTTTGGTAGAGGTGGCTTCG	*Nar*I

*ILV5*	Forward	*TCTACTTTTTACAACAAATATAAAACA*ATGTTGAGAACTCAAGCCGC	

	Reverse	AAAAAA**GGCGCC**ATTCGCGTTTCGGTTCTTGT	*Nar*I

*ILV6*	Forward	*TCTACTTTTTACAACAAATATAAAACA*ATGCTGAGATCGTTATTGCA	

	Reverse	AAAAAA**GACGTC**AACATCCCAATATCCGTCCA	*Aat*II

*BAT2*	Forward	*TCTACTTTTTACAACAAATATAAAACA*ATGACCTTGGCACCCCTAGA	

	Reverse	AAAAAA**GGCGCC**GAATTGTCTTGAGTTGCTTCTAAGGTA	*Nar*I

q*ILV2*	Forward	TCCAAGGTTGCCAACGACACAG	

	Reverse	TGTTGAGCAGCCCACATTTGATG	

q*ILV3*	Forward	TTGCACCTCCACCTCGTTACAC	

	Reverse	ACCGTTGGAAGCGTTGGAAACC	

q*ILV5*	Forward	TTACGCCGTCTGGAACGATGTC	

	Reverse	GAACCAATGGCAACGGCCAAAG	

q*ILV6*	Forward	TACCATGGTGCGTTGCAGTTCC	

	Reverse	AGGTCTTGTTGCGTGTCTGTGC	

q*BAT2*	Forward	GAAATCGGCTGGAAAGGCGAAC	

	Reverse	CTTTGGCCAATGGACCGGTTTG	

q*ACT1*	Forward	TGGATTCTGAGGTTGCTGCTTTGG	

	Reverse	ACCTTGGTGTCTTGGTCTACCG	

^a^Restriction sites used for cloning are shown in bold. The reverse and complementary sequences used for fusion PCRs are shown in italics. The primers for q*ILV2 *q*ILV3 *q*ILV5 *q*ILV6 *q*BAT2 *and q*ACT1 *amplifications were used for quantitative real-time polymerase chain reactions.

The generated blunt end fusion PCR products, PGK1+ILV2, PGK1+ILV3, PGK1+ILV5, PGK1+ILV6 or PGK1+BAT2, were cloned into the pCR-Blunt II-TOPO vector by using the Zero Blunt TOPO PCR Cloning Kit (Invitrogen, Life Technologies, Paisley, UK). The resulting plasmids were designated pTOPO_P+ILV2, pTOPO_P+ILV3, pTOPO_P+ILV5, pTOPO_P+ILV6, and pTOPO_P+BAT2, respectively. The *PGK1 *promoter together with the target gene were cut out from these pTOPO clones, and ligated into vector YDp-L, YDp-W, YDp-H, YDp-U, and YDp-U [13], respectively. The resulting plasmids were designated YDp-L_P+ILV2, YDp-W_P+ILV3, YDp-H_P+ILV5, YDp-U_P+ILV6 and YDp-U_P+BAT2, respectively (Table 3). They were digested with *Bgl*II, *Bgl*II, *Eco*RI, *Nar*I and *Eag*I in the middle of gene *ILV2, ILV3, ILV5, ILV6 *and *BAT2*, respectively. The linearised plasmids were chromosomally integrated into yeast strain CEN.PK 2-1C (a kind gift of Peter Kötter, Goethe Universität Frankfurt, Frankfurt, Germany) in various combinations. The strain with plasmids YDp-L_P+ILV2, YDp-W_P+ILV3 and YDp-H_P+ILV5 integrated in the genome was designated ILV235_XCY561. The strain with plasmids YDp-L_P+ILV2, YDp-W_P+ILV3, YDp-H_P+ILV5 and YDp-U_P+ILV6 was designated ILV2356_XCY605. Two identical strains, independently isolated in the last step of construction, with plasmids YDp-L_P+ILV2, YDp-W_P+ILV3, YDp-H_P+ILV5 and YDp-U_P+BAT2 were designated ILV235BAT2_XCY715 and ILV235BAT2_XCY723, respectively. The genomic integration of the plasmid was confirmed by PCR amplifying PGK1+ILV2, PGK1+ILV3, PGK1+ILV5, PGK1+ILV6 or PGK1+BAT2 fragment from the genomic DNA of the constructed overexpression strains.

### Quantitative real-time PCR

Quantitative real-time PCR was used for quantifying the copy number of the integrated gene and its transcriptional level in the overexpression strain.

### Total genomic DNA isolation

The overexpression strains and the reference strain were cultivated with shaking in 10 ml YPD medium at 30°C overnight. The cells were harvested and lysed with 0.5 mm acid-washed glass beads in 200 μl of breaking buffer through 3 min of vortexing. The breaking buffer contained 2% Triton X-100, 1% SDS, 100 mM NaCl, 100 mM Tris-Cl and 1 mM EDTA with pH at 8.0. Then, 200 μl 1 × Tris/EDTA (TE) buffer, which contained 10 mM Tris-HCl and 1 mM EDTA, was added to the lysed cells. After centrifugation at 12,000 *g *for 5 min, the supernatant was transferred into a new Eppendorf tube. The DNA in the supernatant was precipitated with 1 ml of 100% ethanol, washed with 1 ml of 70% ethanol twice, and resuspended in 50 μl sterile Milli-Q water. The quality and quantity of the isolated genomic DNA was measured using NanoDrop ND-1000 (Thermo Fisher Scientific, Vantaa, Finland). The isolated gDNA with good purity and appropriate quantity was used as template for quantitative real-time PCR to determine the copy number of the integrated genes in the overexpression strains.

### Total RNA isolation

The overexpression strains and the reference stain were cultivated aerobically in mineral medium in shake flasks. Cells were harvested when the optical density of the culture was between 4 and 6 at 600 nm. Total RNA was isolated using Tri Reagent LS from Sigma-Aldrich (St Louis, MO, USA) as recommended by manufacturer. The quality and quantity of the isolated RNA was measured using NanoDrop ND-1000 (Thermo Fisher Scientific, Vantaa, Finland). Then DNase treatment was performed to the RNA samples by using deoxyribonuclease I from Sigma-Aldrich (St Louis, MO, USA). The cDNA was made using M-MuLV RNase H^+ ^reverse transcriptase and random hexamer primer set provided by DyNAmoTM SYBR Green 2-Step qRT-PCR Kit from Finnzymes (Thermo Fisher Scientific, Vantaa, Finland). The negative control of reverse transcription was carried with the RNA, which was not treated by DNaseout. The cDNA and the negative controls were used as the template for quantitative real-time PCR to determine the transcriptional level of the target genes in the overexpression strains and the reference strain.

The primers used for amplifying the small parts of *ILV2, ILV3, ILV5, ILV6, BAT2*, and the reference gene *ACT1*, are listed in Table 3. The resulting PCR fragment sizes were 80, 74, 72, 76, 73 and 125 bp, respectively. The program used for quantitative PCR reactions was as follows: initial denaturation at 95°C for 15 min; 40 cycles of denaturation at 94°C for 10 s, annealing at 66°C for 30 s, fluorescence data collection, extension at 72°C for 30 s; final extension at 72°C for 10 min; melting curve from 65 to 95°C. The threshold cycles (C(t)s) were calculated with the set threshold value by using Mx3005P software (Agilent Technologies, Santa Clara, CA). The ΔΔC(t) method was applied for the relative quantification of the copy numbers and the transcription of *ILV2, ILV3, ILV5, ILV6*, and *BAT2 *in the overexpression strains with respect to the reference strain.

### Fermentation

All anaerobic batch fermentations were performed in triplicate in 2-l fermenters (Braun-Biostat B2, stirred tank, Braun Biotech, Melsungen, Germany) with a working volume of 1.5 l. The fermenter was inoculated with a preculture from a shake flask to an optical density of 0.05 at 600 nm. During cultivation the temperature was maintained at 30°C, agitation was set at 200 rpm, and sparging was kept at 1 l of pure nitrogen per min. pH was maintained at 5.0 by automatic addition of 2 N NaOH. The CO_2 _and O_2 _concentrations in the effluent gas from the fermenter were analysed by gas monitor (Innova 1311, Innova Airtech Instruments, Denmark). Samples were taken throughout the cultivation for optical density measurement, biomass dry weight determination, and high-performance liquid chromatography (HPLC) analysis. Samples for GC analysis were taken when glucose was depleted (defined by the zero value of CO_2 _in the effluent gas).

### Analytical methods

Optical density was measured at 600 nm with a Shimadzu UV-Mini 1240 spectrophotometer, (Shimadzu Scientific Instruments, Kyoto, Japan). Dry weight of biomass was determined gravimetrically by filtration of a known volume of culture over a predried and weighed 0.45 μm-pore-size filter. The filter with biomass was rinsed with a twofold volume of 0.9% NaCl, dried in a microwave oven at 150 W for 20 min and weighed again.

The concentrations of glucose, pyruvate, acetate, succinate, glycerol, and ethanol were measured by using isocratic high-performance liquid chromatography with refractive Index detection on an Agilent 1100 system (Agilent Technologies, Waldbronn, Germany) equipped with cooled autosampler (5°C). Separation was conducted on an Aminex HPX-87H ion-exclusion column, and 5 mM of H_2_SO_4 _as the mobile phase at a flow rate of 0.6 ml/min at 60°C was used. In all, 20 μl of sample was injected, and concentrations were determined against freshly prepared external standards.

Isobutanol was determined using static head space-gas chromatography on a PerkinElmer HS 40 head space sampler (PerkinElmer, Waltham, MA, USA) connected to a Perkin Elmer PE Autosystem gas chromatograph (PerkinElmer, Waltham, MA, USA) equipped with a Flame Ionization Detector and 60 m, 0.25 mm ID, 1 μm, DB-5 capillary column. Samples (1 ml in 20 ml sealed head space vials) were equilibrated for 5 min at 60°C in the auto sampler. The temperature of the needle and the transfer line was 90°C. Helium carrier gas was used at 20 psi. The temperatures of the injector and the detector were set to 200°C and 250°C, respectively. The oven temperature program was 1 min at 60°C and then 10°C per min to 150°C.

Quantification had to be performed using standard addition, since the slope of the calibration curve in pure water was different from the one obtained by spiking the fermentation sample with standards. Standard addition was performed at three different levels using 50 μl isobutanol standard solutions to certain volume of sample solutions. The corresponding addition were 7% to 100%, 40% to 150%, and 57% to 300% extra isobutanol. The R^2 ^values of the corresponding addition curves were in all cases larger than 0.96, and in most cases over 0.98. The initial isobutanol concentration was extrapolated by the readings changed before and after adding the standard solutions. Quantification of 2-methylbutanol and 3-methylbutanol was performed by using isobutanol as the internal standard. The response factor difference of 2-methylbutanol and 3-methylbutanol versus isobutanol were determined by calibration with mixtures of isobutanol, 2-methylbutanol and 3-methylbutanol spiked into the media blank, at different concentrations similar to the ones observed in real samples.

## Competing interests

The authors declare that they have no competing interests.

## Authors' contributions

XC participated in design of the study, did all experimental work and participated in writing the manuscript. KFN designed the isobutanol analysis method and participated in writing the corresponding parts of the manuscript. IB participated in writing the manuscript. MCKB participated in design of the study and participated in writing the manuscript. KK participated in design of the study and in writing of the manuscript. All authors read and approved the final manuscript.
